# Digital trust in medical tourism portals: multiple pathways to consumer confidence

**DOI:** 10.3389/fdgth.2026.1885464

**Published:** 2026-07-30

**Authors:** Vinaytosh Mishra

**Affiliations:** 1 Gulf Medical University, Ajman, United Arab Emirates; 2 Datta Meghe Institute of Higher Education and Research Deemed to be University, Wardha, India

**Keywords:** configurational pathways, consumer confidence, digital platforms, fuzzy-set qualitative comparative analysis (fsQCA), medical tourism portals

## Abstract

This study examines how consumer confidence is configurationally constructed in medical tourism portals using fuzzy-set Qualitative Comparative Analysis (fsQCA). Drawing on established theoretical perspectives, seven antecedent conditions are analyzed: Information Quality, Trust and Credibility Signals, Platform Quality and Usability, Security and Privacy Protection, Risk Perception Reduction, Communication and Responsiveness, and Governance and Ethical Transparency. Survey data from 163 portal users were analyzed across 128 possible configurations, applying a consistency threshold of 0.85 and a frequency cutoff of 3. The necessary condition analysis indicates that no single factor meets the 0.90 consistency threshold, suggesting the absence of a dominant driver of consumer confidence. Sufficiency analysis identifies four distinct equifinal pathways explaining 60% of high-confidence cases, with an overall solution consistency of 0.77. The findings highlight compensatory and asymmetric mechanisms in which combinations of informational, technological, and institutional factors jointly shape consumer confidence in digital healthcare platforms.

## 1 Introduction

**Figure 1:**
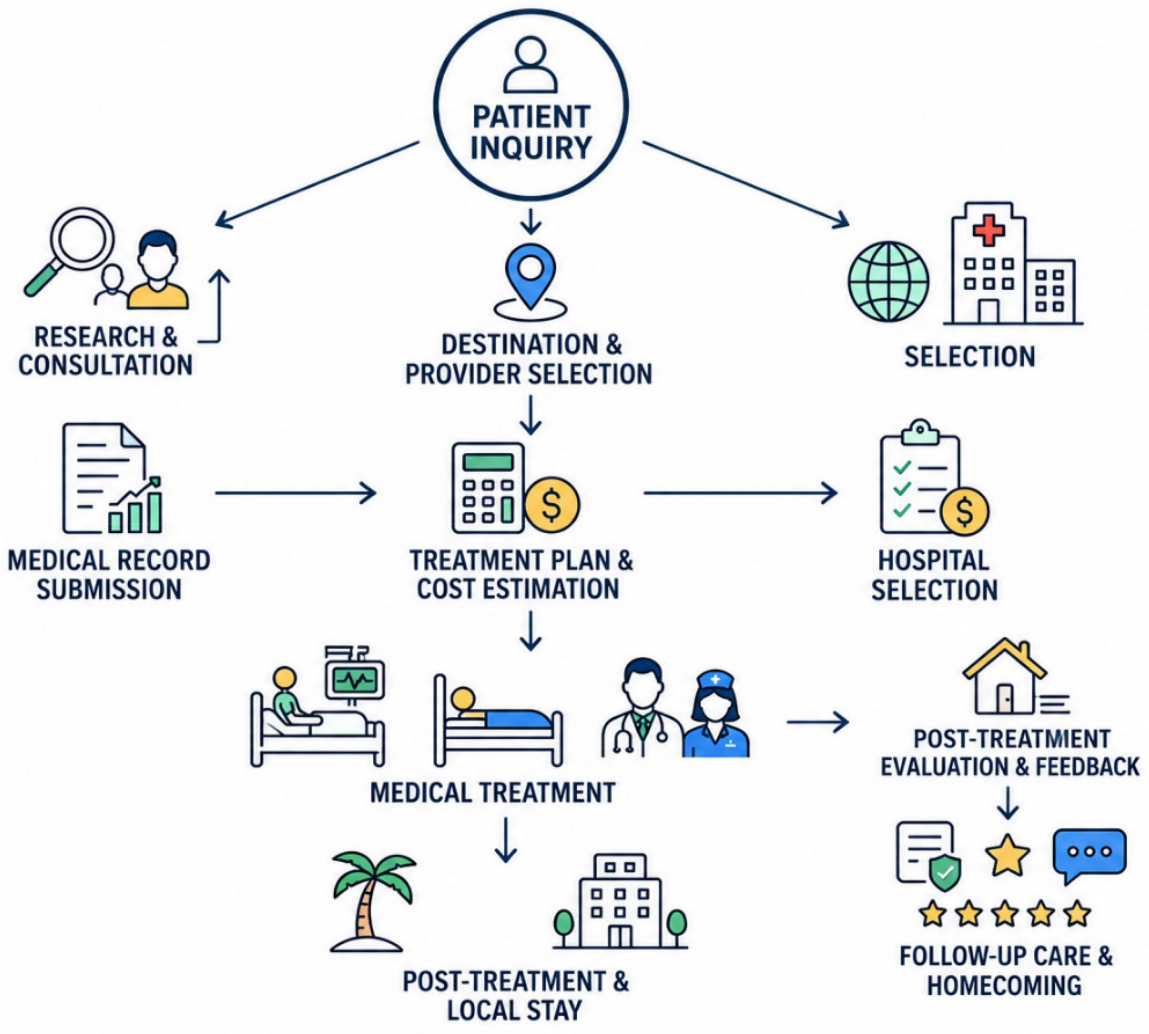
Typical medical tourism process.

The global medical tourism sector has undergone rapid digitalization, transforming how patients search for, evaluate, and access international healthcare services (1). Recent analyses highlight that medical tourism is evolving into a highly interconnected ecosystem where digital platforms serve as intermediaries linking patients, hospitals, facilitators, and insurers (2). These platforms streamline information exchange, reduce search costs, and enhance transparency in treatment options, pricing, and provider credibility (3, 4). A typical medical tourism process is depicted in Figure 1. Studies show that the growth of this industry is closely linked to real-world pressures people face every day, such as rising healthcare costs in developed countries, long waiting times for treatment, and the increasing ease of global travel (5). More patients are looking beyond borders for timely and affordable care. Digital platforms are making this transition smoother by helping patients find trusted providers, arrange teleconsultations, and manage their entire treatment journey from initial inquiry to follow-up care in one coordinated experience (6).

### 1.1 Digital platforms in medical tourism

Healthcare has witnessed increased digitalisation in the recent past (7). Digital platforms, whether simple e-health apps or AI-powered medical tourism marketplaces, have become vital companions for patients seeking care abroad. For many international patients, these tools are not just websites; they are trusted guides that help them make informed and confident decisions (8). Today, patients can speak with doctors online before ever boarding a flight, allowing them to understand their treatment options and assess whether traveling is the right choice. Medical records can be securely shared between doctors at home and providers overseas, ensuring continuity of care and reducing uncertainty. Clear pricing information and quality comparisons help patients avoid unpleasant surprises and make cost-effective decisions. Patient reviews and trust indicators also play an important role, offering reassurance through the experiences of others who have walked the same path (3). Overall, e-health applications have made global healthcare more accessible and transparent. They empower patients to understand complex medical information, weigh risks carefully, and coordinate appointments, travel, and follow-up care with greater ease. In doing so, these digital tools reduce the stress and uncertainty often associated with seeking treatment across borders.

### 1.2 Characteristics of medical travel

Medical travel remains high-risk and high-uncertainty. Clinically, cross-border procedures occur in diverse regulatory environments with uneven accreditation and liability protections, underscoring that accreditation signals help but do not guarantee safety (9, 10). Information asymmetry persists; verifying credentials, outcomes, and costs remains difficult amid nonstandardized reporting (11, 12). Logistical and post-operative risks stem from disrupted continuity of care and limited follow-up after returning home, along with travel-related recovery complications (11). Digital platforms mitigate some uncertainty via verified provider networks, AI risk assessments, and secure data sharing, yet cross-border complexity and regulatory variability limit the elimination of risk (10, 11). Policy and practice implications emphasize credible accreditation, transparent outcomes and pricing, explicit continuity-of-care arrangements, and harmonized data sharing, while acknowledging residual uncertainty inherent to cross-border. Globally, emerging countries' experiences illustrate benefits and equity concerns, including impacts on local systems and the need for governance to protect patients and host populations (13).

### 1.3 Research motivation and problem statement

Despite the rapid growth of digital platforms in medical tourism, building consumer confidence remains a significant challenge. Unlike conventional online purchases, medical tourism decisions involve substantial financial commitments, potential clinical risks, legal uncertainties, and concerns regarding continuity of care across national borders. Patients are often required to make complex healthcare decisions based primarily on information available through digital platforms without having direct experience of the healthcare provider, destination, or treatment environment. Although medical tourism portals increasingly provide information regarding hospitals, physicians, treatment packages, accreditation status, and patient testimonials, consumers frequently face difficulties in assessing the credibility, completeness, and reliability of such information. Concerns regarding data privacy, provider legitimacy, hidden costs, treatment outcomes, and post-treatment follow-up continue to create uncertainty and may undermine confidence in digital platforms.

Existing research has examined trust in medical tourism primarily through linear approaches that investigate the independent effects of factors such as information quality, website quality, perceived risk, or accreditation. However, trust formation in medical tourism is inherently complex and may not be driven by a single factor. Different consumers may develop confidence through different combinations of informational, technological, institutional, and relational assurances. Consequently, a configurational perspective is needed to understand how multiple conditions interact to create consumer confidence. Addressing this gap is important because digital trust directly influences patients' willingness to use medical tourism portals, engage with international healthcare providers, and ultimately pursue cross-border healthcare services. Understanding the combinations of factors that generate confidence can help platform developers, healthcare providers, and policymakers design more trustworthy and patient-centered digital ecosystems.

### 1.4 Contribution of the study

Although trust has been widely studied in healthcare, tourism, and electronic commerce, several important gaps remain in the medical tourism literature. First, most existing studies examine trust using linear analytical approaches such as regression analysis or structural equation modelling, which assume that individual factors independently influence consumer confidence. Such approaches are useful for identifying net effects but are less capable of capturing the complex interactions among informational, technological, institutional, and relational factors that characterize medical tourism decision-making. Second, prior research has largely focused on isolated determinants of trust, including website quality, accreditation, destination image, service quality, or perceived risk. Consequently, limited attention has been given to understanding how these factors combine and interact to generate consumer confidence. In practice, patients rarely evaluate healthcare providers based on a single criterion; rather, they simultaneously consider information quality, security, governance mechanisms, trust signals, communication support, and perceived risks when making decisions about cross-border healthcare. Third, despite the increasing digitalization of medical tourism, configurational approaches remain scarce in this domain. As a result, little is known about whether different combinations of trust-building mechanisms can lead to the same outcome of consumer confidence, or whether certain factors can compensate for weaknesses in others.

This study addresses these gaps by applying fuzzy-set Qualitative Comparative Analysis (fsQCA) to examine how multiple antecedent conditions jointly contribute to consumer confidence in medical tourism portals. Unlike traditional linear approaches, fsQCA allows the identification of equifinal pathways, conjunctural causation, and compensatory mechanisms. The study, therefore, contributes to the literature by demonstrating that digital trust in medical tourism is not driven by a single dominant factor but emerges through multiple alternative combinations of informational, technological, and institutional assurances. From a practical perspective, the findings provide actionable guidance for portal designers, healthcare providers, destination managers, and policymakers by identifying alternative pathways through which confidence can be strengthened in digital healthcare ecosystems.

## 2 Literature review

### 2.1 Theoretical underpinning

Digital trust can be analysed through the theoretical lenses of various technologies discussed in this section.

#### 2.1.1 Signalling theory

Medical tourism portals operate under information asymmetry, where patients lack direct knowledge of provider quality, safety standards, and procedural outcomes. In such contexts, signals are used to reduce uncertainty and convey credibility (14). According to Signalling Theory, Information Quality (INFQ) functions as a cognitive signal of competence and transparency; Trust & Credibility Signals (TRUS) (e.g., accreditation badges, testimonials, certifications) function as reputational signals, while Governance Transparency (GOVE) serves as an institutional legitimacy signal. When signals are perceived as credible, observable, and costly to imitate, they reduce uncertainty and enhance confidence. However, signalling theory alone assumes additive effects (15). The present study extends this by arguing that signals operate in combinations, not isolation.

#### 2.1.2 Perceived risk theory

Medical tourism inherently involves (1) Clinical risk, (2) Financial risk, (3) Legal/regulatory risk, and (4) post-operative continuity risk (16). Perceived Risk Theory posits that consumers seek mechanisms to reduce uncertainty before committing to high-stakes decisions (17). Within this framework, Risk Perception Reduction (RISK) directly lowers perceived uncertainty, Security & Privacy Protection (SECP) mitigates data-related risk, and Information Quality (INFQ) reduces ambiguity about procedures and outcomes. In cross-border healthcare, perceived risk is multidimensional and cannot be fully eliminated. Therefore, consumer confidence emerges when portals provide visible, structured risk-mitigation mechanisms.

#### 2.1.3 Institutional trust theory

Institutional Trust Theory argues that trust in high-uncertainty environments is often transferred from institutional structures rather than interpersonal familiarity (18). In medical tourism portals, accreditation frameworks, regulatory alignment, complaint mechanisms, and transparent governance structures serve as institutional safeguards (19). These reduce reliance on personal familiarity and create what is known as system-based trust. This perspective is particularly relevant in cross-border healthcare, where regulatory regimes vary, legal recourse may be unclear, and cultural expectations differ. Institutional structures, therefore, become critical substitutes for direct relational trust. In medical tourism, institutional trust extends beyond individual hospitals and digital platforms to encompass broader health system characteristics, including regulatory effectiveness, healthcare quality standards, accreditation regimes, patient safety governance, and continuity-of-care mechanisms. These system-level attributes provide important institutional assurances that influence consumer confidence in cross-border healthcare decisions.

#### 2.1.4 Socio-technical systems theory

Medical tourism portals are not purely technological platforms; they are socio-technical systems integrating technological architecture (usability, security, interface design), organizational governance, institutional frameworks, and user perceptions. Socio-Technical Systems Theory posits that outcomes emerge from the interaction between technical and social components, not from any single element (20). Accordingly, Usability (USAB) reflects experiential system quality, and Security (SECP) reflects infrastructural robustness. Communication (COMM) reflects relational interface dynamics, and Governance (GOVE) reflects institutional embedding (21). Consumer confidence (CONF) thus emerges from the alignment of these interacting subsystems.

#### 2.1.5 Configurational theory of causality

Traditional trust research often assumes linear, symmetric relationships between predictors and outcomes. However, this study adopts a configurational causality perspective, which assumes: Multiple pathways can lead to high consumer confidence (Equifinality), Conditions operate in combinations (Conjunctural Causation), and the causes of high confidence differ from the causes of low confidence (Causal Asymmetry) (22). This theoretical stance aligns directly with the fsQCA methodology described in the manuscript.

Recent studies increasingly employ fsQCA to investigate complex socio-technical phenomena in healthcare, digital services, and platform ecosystems where outcomes emerge through multiple interacting conditions rather than single independent predictors. The method is particularly suitable for examining digital trust because trust formation often involves equifinality, causal asymmetry, and compensatory mechanisms among informational, technological, institutional, and relational factors. Consequently, fsQCA provides an appropriate analytical framework for understanding how different combinations of trust-building mechanisms generate consumer confidence in medical tourism portals. The summary of theoretical integration in the study is depicted in Table 1.

**Table 1:** Summary of theoretical integration in the study.

Theoretical Lens	Explains	Constructs
Signalling Theory	Reduction of information asymmetry	INFQ, TRUS
Perceived Risk Theory	Uncertainty reduction	RISK, SECP
Institutional Trust Theory	System-based trust formation	GOVE
Socio-Technical Systems Theory	Interaction of technical & social elements	USAB, COMM, SECP
Configurational Theory	Complex, non-linear causality	fsQCA modeling

The five theoretical perspectives are complementary and collectively explain the formation of consumer confidence in medical tourism portals. Signalling Theory explains how information quality and credibility cues reduce information asymmetry; Perceived Risk Theory explains how security and risk-mitigation mechanisms reduce uncertainty; Institutional Trust Theory highlights the role of governance structures and health system assurances; and Socio-Technical Systems Theory emphasizes the interaction between technological features and social processes. Configurational Theory integrates these perspectives by recognizing that consumer confidence does not emerge from any single mechanism but from different combinations of informational, technological, institutional, and relational conditions. Accordingly, the study adopts a configurational approach to examine how these theoretically grounded factors jointly contribute to consumer confidence in medical tourism portals.

### 2.2 Digital trust in medical tourism

Digital trust is pivotal in medical tourism, shaping patient decisions, perceived safety, and ongoing engagement with providers across borders. Trust in digital platforms emerges from credible online information, transparent outcomes, and secure data handling, which collectively reduce perceived risk in high-uncertainty purchases (23). Online reviews and digital promotion amplify trust and perceived value, thereby strengthening intention to seek care abroad, especially when they translate into tangible benefits and reliable patient experiences (19, 23, 24). Simultaneously, stakeholders such as physicians, nurses, and destination marketers recognize that trust fractures can arise from information gaps, perceived inequities, or opaque treatment pathways, underscoring the need for third-party validation and robust governance of cross-border care (16, 25). Emerging technologies such as blockchain and trusted-review systems offer pathways to disintermediation, transparency, and secure data exchange, further reinforcing digital trust in medical tourism contexts (23, 26). Overall, cultivating credible, patient-centered digital ecosystems is essential for sustainable trust in medical tourism (23, 24).

Recent developments in digital health have further expanded the concept of trust beyond traditional website credibility and information quality. AI-enabled healthcare platforms increasingly support teleconsultations, clinical decision support, personalized treatment recommendations, and patient engagement across the care continuum (27). As healthcare interactions become more digitally mediated, trust is influenced not only by provider reputation but also by perceptions of algorithmic transparency, data privacy, platform governance, and technological reliability (28). Similarly, platform governance mechanisms such as provider verification, accreditation oversight, dispute resolution processes, and ethical data management play an important role in maintaining confidence in digital health ecosystems (29). These developments suggest that trust in medical tourism portals is embedded within broader digital health infrastructures that combine technological, institutional, and governance-based assurances.

### 2.3 Factors affecting digital trust

This literature review synthesizes evidence on how information quality, trust signals, usability, security, risk mitigation, communication, and governance contribute to high consumer confidence in medical tourism portals. Drawing on a diverse set of sources across medical tourism, tourism information systems, health informatics, and online trust research, we identify how each factor supports informed decision-making, perceived safety, and trust in portals that help consumers compare providers, procedures, and destinations. Where sources disagree or offer nuanced views, the synthesis highlights these tensions and points to areas where further empirical work is warranted.

#### 2.3.1 Information quality (INFQ)

High-quality information on medical tourism portals supports informed choice, reduces perceived risk, and enhances trust, thereby boosting consumer confidence. Portals that present accurate, current, and comprehensive information about destinations, facilities, procedures, accreditations, and provider capabilities are associated with improved decision-making and perceived reliability (30, 31). The literature on medical tourism emphasizes that information on quality, safety, ethics, and accreditation is central to consumer confidence; transparent, evidence-based content underpins trust in destinations and providers (32, 33). Relationship marketing research in medical tourism indicates that information transparency and credible content influence consumer trust and behavioural intentions toward the destination (34). Some studies explicitly address content strategy (e.g., tailoring information to cultural contexts) to enhance trust through perceived informational sufficiency and relevance (33).

#### 2.3.2 Trust and credibility signals (TRUS)

Trust signals embedded in portals (accreditations, testimonials, expert endorsements, and transparent outcome data) are critical to consumer confidence in medical tourism decisions. Trust in medical tourism is linked to trust in healthcare organizations and the online information environment; trust signals such as accreditation (NABH, JCI), credentialed providers, and transparent patient outcomes bolster confidence (31, 32). Studies on online health information stress that trust features of websites (badges, credentialing, quality signals) correlate with consumer trust and willingness to engage (33). Relationship marketing literature connects trust, satisfaction, and loyalty, mediated by credible information and consistent trust signals across the portal and partner networks (34). Cultural framing and content customization can modulate how users from different cultural backgrounds interpret trust signals, suggesting a need for adaptive trust-signalling strategies.

#### 2.3.3 Platform quality and usability (USAB)

The usability of medical tourism portals affects user experience, navigation efficiency, perceived information quality, and ultimately consumer confidence. Usability evaluation studies of tourism and health portals show that intuitive navigation, clear information architecture, and task-focused usability contribute to positive user experiences and perceived reliability (30, 31). Usability testing using established heuristics demonstrates that site usability directly influences trust perceptions and perceived credibility, which in turn shapes confidence in medical tourism decisions (31). Studies on tourism portals emphasize usability as a determinant of user satisfaction and engagement with information systems, reinforcing the link to consumer confidence (35). The medical tourism domain uniquely combines usability with sensitive decision content; portals should prioritize goal-oriented workflows, readability, and transparent pathways for procedural information to foster confidence (31).

#### 2.3.4 Security and privacy protection (SECP)

Security and privacy protections are foundational to consumer confidence in medical tourism portals. These conditions are repeatedly highlighted as critical to credibility; breaches or perceived privacy risks undermine trust in health-related portals (and by extension in medical travel decisions). Scholarly work on health portals and medical information underlines the importance of data confidentiality, access controls, and privacy safeguards for maintaining user trust and engagement (31). Security is frequently paired with usability (privacy-friendly design) and trust signals; portals should implement robust data protection measures and communicate them clearly to users (36).

#### 2.3.5 Risk perception reduction (RISK)

Proactive risk mitigation (clear disclaimers, quality and safety criteria, consent processes, and transparent procedure-related risks) reduces perceived risk and enhances confidence. Nursing and ethics-focused perspectives emphasize safety, quality criteria, and regulatory considerations when accessing medical services abroad; clear risk information contributes to informed consent and confidence (37). The broader medical tourism literature discusses patient safety, consent, and ethical considerations as central to credible portal information and provider selection (32). Technical and marketing literature on risk communication stresses that transparent risk disclosure and management strategies build trust and reduce perceived uncertainty in online health marketplaces (33). Diverging views exist on the magnitude of risk across destinations; portals should present standardized risk disclosures and compare safety/quality metrics across providers to support confidence-building.

#### 2.3.6 Communication and responsiveness (COMM)

Effective communication strategies (clear language, multilingual content, culturally tailored messaging, and timely updates) bolster consumer confidence in medical tourism portals. Cross-cultural trust research shows that Hofstede-informed content customization can influence trust-building through content that matches cultural expectations and communication styles (33, 38). Social media and online communication research advocate fostering trust through content strategies aligned with cultural nuances, testimonials, and transparent information sharing; this is linked to consumer confidence in medical tourism contexts (33). Relationship marketing literature underscores the role of communication in shaping expectations, satisfaction, and loyalty; consistent, clear, and responsive communication enhances user confidence (34). Communication is especially salient for international users; portals should support multilingual interfaces, culturally aware content, and timely responses to inquiries and feedback.

#### 2.3.7 Governance and ethical transparency (GOVE)

Strong governance structures (accreditation requirements, regulatory alignment, quality assurance, and partner governance) underpin consumer confidence in medical tourism portals. Governance-related discussions emphasize international accreditation, standards, and ethical considerations as foundational to trust in medical tourism and related portals (32, 38). Recent studies highlight the role of healthcare facilities and governance in shaping medical tourism success and destination competitiveness; governance quality affects perceived provider quality and decision confidence (32, 39). Strategic and policy-oriented literature discusses government-led branding, portals, and public-private collaborations (e.g., Heal in India, UAE DXH) as governance mechanisms that enhance credibility and destination appeal (33, 40). Governance signals should be embedded into portal design as visible policy alignment, accreditation badges, provider verification processes, and transparent dispute resolution mechanisms to sustain confidence over time. The summary of the conditions leading to digital trust is summarised in Table 2.

**Table 2:** Conditions used in the configurational digital trust model.

Condition	Brief Description	Citing Literature
Information Quality **(INFQ)**	Accuracy, completeness, clarity, and timeliness of medical, procedural, pricing, and accreditation information provided on the portal enhance informed decision-making and reduce uncertainty.	(30, 31, 33, 34)
Trust & Credibility Signals **(TRUS)**	Presence of accreditation badges, verified credentials, testimonials, outcome transparency, and professional presentation that signal legitimacy and reliability.	(31, 32, 34)
Platform Quality & Usability **(USAB)**	Ease of navigation, comparison tools, intuitive interface, performance speed, and overall user experience shape perceived credibility and satisfaction.	(30, 35)
Security & Privacy Protection **(SECP)**	Data confidentiality, secure transactions, privacy controls, transparent data policies, and visible security assurances.	(31, 36)
Risk Perception Reduction **(RISK)**	Provision of clear risk disclosures, safety criteria, cost transparency, consent mechanisms, and follow-up care assurances to reduce perceived uncertainty.	(32, 33, 38)
Communication & Responsiveness **(COMM)**	Multilingual support, timely responses, accessible contact channels, culturally tailored communication, and interactive support systems.	(33, 34, 37)
Governance & Ethical Transparency **(GOVE)**	Regulatory alignment, accreditation oversight, complaint mechanisms, ethical commitments, and visible institutional compliance.	(32, 38, 40)
Consumer Confidence **(CONF)**	Overall, digital trust, assurance, and willingness to use or book through the medical tourism portal.	(19, 23, 24, 26)

Prior studies often conceptualize trust as a linear, additive construct if individual predictors independently shape user confidence in online services (19, 41). However, digital trust within medical tourism ecosystems is inherently multi-dimensional and non-linear, influenced by complex interactions among platform quality, risk perceptions, and cross-border healthcare uncertainties. Despite the growing importance of digital platforms in medical tourism, a significant gap remains in understanding how consumer confidence is formed. Most prior studies examine the independent effects of factors such as information quality, website usability, accreditation, and perceived risk. However, medical tourism decisions are complex and involve the simultaneous evaluation of multiple informational, technological, institutional, and relational cues. Consequently, existing research provides limited insight into how these factors interact to generate consumer confidence. A configurational perspective is therefore needed to examine how different combinations of trust-building mechanisms shape confidence in high-risk, high-uncertainty healthcare decisions. This understanding is important for both theory and practice in the rapidly evolving medical tourism ecosystem. With this background, the research question for this study is:

RQ: What configurations of information quality, trust signals, usability, security, risk mitigation, communication, and governance lead to high consumer confidence in medical tourism portals?

To address this research question, the study has three research objectives:

1. Identify the conditions leading to consumer confidence in the medical tourism portal.
2. To examine whether any single condition constitutes a necessary condition for achieving high consumer confidence.
3. To identify and analyse the configurational pathways through which combinations of different identified conditions lead to high consumer confidence in medical tourism portals.

## 3 Research methodology

This study employs fuzzy-set Qualitative Comparative Analysis (fsQCA) to identify configurational pathways leading to consumer confidence in medical tourism portals. fsQCA was selected because consumer confidence in medical tourism portals is expected to emerge from multiple interacting conditions rather than a single dominant predictor. The method is particularly suitable for examining equifinality, causal complexity, and compensatory relationships among trust-building mechanisms. fsQCA is grounded in set theory and Boolean algebra, allowing causal conditions to be expressed as sets and relationships to be evaluated in terms of subset and superset relations (42). Unlike regression-based approaches that estimate net effects under assumptions of linearity and symmetry, fsQCA assumes (1) equifinality (multiple pathways leading to the same outcome), (2) conjunctural causation (conditions operate in combination), and (3) causal asymmetry (presence and absence of outcome may have different explanations) (22). Let Y = Consumer Confidence in Medical Tourism Portal and $X_{1}$, $X_{2}$, …, $X_{k}$ = Causal conditions (technical, organizational, societal, individual factors). The objective is to identify configurations $f(X_{1},X_{2},\ldots ,X_{k})\to Y.$

The research process consisted of five sequential stages. First, constructs affecting digital trust in medical tourism portals were identified through a comprehensive literature review. Second, a structured questionnaire was developed and administered to healthcare portal users. Third, responses were aggregated and calibrated into fuzzy-set membership scores. Fourth, necessary conditions and truth-table analyses were conducted. Finally, Boolean minimization was applied to identify sufficient configurational pathways leading to consumer confidence. This structured process ensured methodological rigor and alignment between the theoretical framework and empirical analysis.

### 3.1 Survey instrument development

The survey instrument was developed through a review of the literature on digital trust, medical tourism, healthcare information systems, and online platform adoption. The questionnaire was structured around the seven antecedent conditions identified in the conceptual framework: Information Quality (INFQ), Trust and Credibility Signals (TRUS), Platform Quality and Usability (USAB), Security and Privacy Protection (SECP), Risk Perception Reduction (RISK), Communication and Responsiveness (COMM), Governance and Ethical Transparency (GOVE), and the outcome condition Consumer Confidence (CONF). Multiple items were developed for each construct based on themes identified in prior literature and adapted to the context of medical tourism portals. All items were measured using a five-point Likert scale ranging from strongly disagree (1) to strongly agree (5). Construct scores were calculated by averaging the corresponding item responses before calibration into fuzzy-set membership scores.

### 3.2 Calibration of fuzzy sets

Raw survey scores were transformed into fuzzy-set membership scores ranging from 0 (full non-membership) to 1 (full membership). Each condition was calibrated using three qualitative anchors: (1) full membership threshold: $e_{1}$ (2) Crossover point: *c*, and (3) full non-membership threshold: $e_{0}$. Using Ragin's direct calibration method, fuzzy membership was computed through a log-odds transformation:

$$\mu (x_{i})=\frac{1}{1+\exp[-\alpha (x_{i}-c)]}$$

where: $x_{i}$ = raw score; c = crossover point; and α = scaling factor determined by the distance between anchors. This transformation ensures that:

$$\mu (x_{i})\in [0,1]$$

where $\mu (x_{i})$ represents the degree of membership in the set. This study uses a five-point Likert scale to measure respondents' perceptions across the identified conditions. Construct scores were obtained by averaging the corresponding item responses before calibration. Because established substantive calibration thresholds for digital trust constructs in medical tourism portals are currently unavailable, an empirical calibration approach was adopted. Following common practice in exploratory fsQCA applications, the observed maximum value, mean value, and minimum value were used as anchors for full membership, crossover, and full non-membership, respectively. This approach preserves the empirical distribution of the data while allowing respondents to be positioned relative to the observed range of perceptions. Although theoretical calibration is generally preferred when well-established substantive thresholds exist, empirical calibration was considered appropriate given the exploratory nature of the study and the absence of accepted calibration standards for the constructs examined.

### 3.3 Set-theoretic operations

In fuzzy-set logic, logical operators are defined as:

Conjunction (AND)

$$\mu (X\cap Z{)}_{i}=\min(\mu (X_{i}),\mu (Z_{i}))$$

Disjunction (OR)

$$\mu (X\cup Z{)}_{i}=\max(\mu (X_{i}),\mu (Z_{i}))$$

Negation (NOT)

$$\mu (∼X{)}_{i}=1-\mu (X_{i})$$

These operators allow the construction of configurational expressions such as:

$$X_{1}\cdot X_{2}\cdot ∼X_{3}\to Y$$

### 3.4 Necessary conditions analysis

A condition *X* is necessary for the outcome *Y* if:

Y⊆X

Empirically, necessity is evaluated using the consistency metric:

$$\mathrm{Consistenc}y_{\mathrm{nec}}(X\leq Y)=\frac{\sum_{i}\min(\mu (X_{i}),\mu (Y_{i}))}{\sum_{i}\mu (Y_{i})}$$

A consistency score ≥0.90 was used as the threshold for identifying necessary conditions.

Coverage for necessity was calculated as:

$$\mathrm{Coverag}e_{\mathrm{nec}}=\frac{\sum_{i}\min(\mu (X_{i}),\mu (Y_{i}))}{\sum_{i}\mu (X_{i})}$$

This indicates the empirical relevance of the necessary condition.

### 3.5 Truth table construction

Next, a fuzzy truth table was constructed to represent all logically possible combinations of k causal conditions. The total number of logically possible configurations equals:

$$2^{k}$$

Each row represents a configuration:

$$C_{j}=X_{1}^{a_{1}}\cdot X_{2}^{a_{2}}\cdot \ldots \cdot X_{k}^{a_{k}}$$

where $a_{m}\in \{0,1\}$ denotes presence or absence.

Each configuration's sufficiency for the outcome was evaluated.

### 3.6 Sufficiency analysis

A configuration *C* is sufficient for the outcome *Y* if:

C⊆Y

Consistency of sufficiency is calculated as:

$$\mathrm{Consistenc}y_{\mathrm{suff}}=\frac{\sum_{i}\min(\mu (C_{i}),\mu (Y_{i}))}{\sum_{i}\mu (C_{i})}$$

Configurations with consistency ≥0.85 were retained.

Next, the Raw coverage is calculated as:

$$\mathrm{Raw}\;\mathrm{Coverage}=\frac{\sum_{i}\min(\mu (C_{i}),\mu (Y_{i}))}{\sum_{i}\mu (Y_{i})}$$

Unique coverage measures the proportion explained exclusively by that configuration.

### 3.7 Boolean minimization

The solution terms were simplified using Boolean algebra, following the Quine–McCluskey minimization procedure. If two configurations differ in only one condition but produce the same outcome. This reduction identifies core causal conditions.

$$X_{1}X_{2}X_{3}+X_{1}X_{2}∼X_{3}=X_{1}X_{2}$$

Final solutions are expressed as follows, indicating multiple equifinal pathways.

$$Y=C_{1}+C_{2}+C_{3}$$

### 3.8 Solution consistency

Overall solution consistency is calculated as:

$$\mathrm{Solution}\;\mathrm{Consistency}=\frac{\sum_{i}\min(\mu (\mathrm{Solutio}n_{i}),\mu (Y_{i}))}{\sum_{i}\mu (\mathrm{Solutio}n_{i})}$$

Overall coverage:

$$\mathrm{Solution}\;\mathrm{Coverage}=\frac{\sum_{i}\min(\mu (\mathrm{Solutio}n_{i}),\mu (Y_{i}))}{\sum_{i}\mu (Y_{i})}$$

These metrics assess the explanatory strength of the combined pathways.

Robustness of the solution was assessed by conducting a series of sensitivity analyses to ensure the stability and reliability of the results. Specifically, consistency thresholds were varied within a reasonable range (0.80–0.90) to examine whether the identified configurational pathways remained stable under different inclusion criteria. Calibration anchors for full membership, crossover, and full non-membership were also adjusted to test the sensitivity of the findings to alternative set specifications. In addition, intermediate and parsimonious solutions were compared to evaluate the extent to which simplifying assumptions influenced the final solution structure. The stability of configurations across these alternative specifications strengthens confidence in the robustness and substantive validity of the identified pathways to Consumer Confidence in Medical Tourism portals.

This study captures the complex, nonlinear, and asymmetric dynamics underlying Consumer Confidence in Medical Tourism Portals. This study models Consumer Confidence (CONF) as given by the following equation.

f(INFQ,TRUS,USAB,SECP,RISK,COMM,GOVE)→Y(CONF)

**Figure 2:**
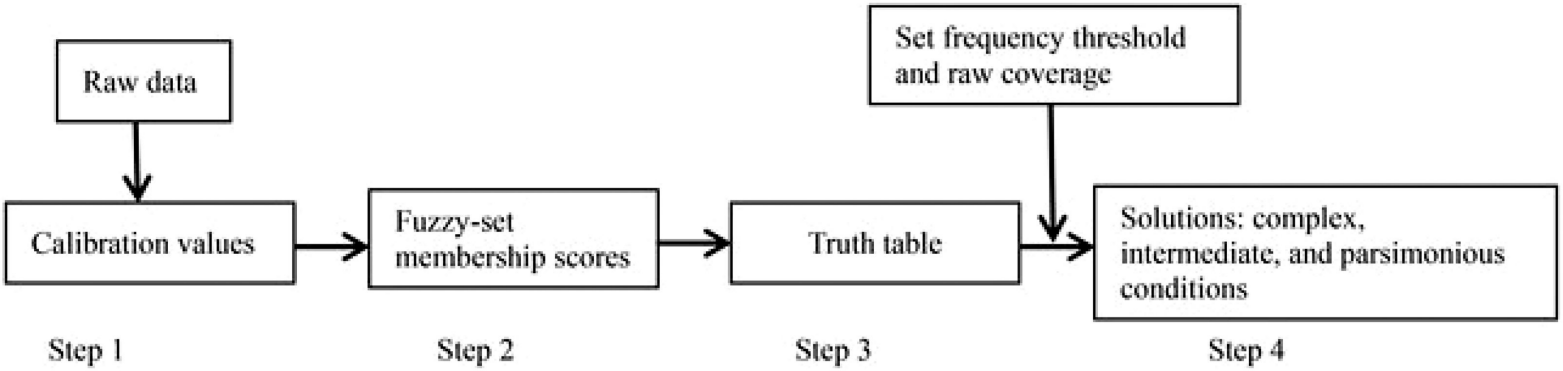
Summary of the fsQCA method used in the study.

The fsQCA approach, therefore, offers a theoretically coherent and empirically rigorous method for identifying actionable pathways in complex socio-technical systems. The summary of the research methodology used in this study is depicted in Figure 2.

### 3.9 Sampling plan

This study employed a purposive, individual-level sampling strategy to examine configurational pathways affecting Consumer Confidence in Medical Tourism Portals. using fsQCA. Each completed questionnaire represented one individual and was provided by a person who has used healthcare portals for information search, not necessarily for a medical tourism motive. This sampling approach was adopted because consumer confidence in medical tourism portals is primarily formed during the information-search and evaluation stages that precede actual travel decisions. Consequently, individuals familiar with healthcare-related digital platforms were considered appropriate respondents for examining trust-building mechanisms associated with information quality, security, usability, communication, governance, and credibility signals. Nevertheless, the findings should be interpreted with caution because perceptions of actual medical tourists may differ from those of general healthcare portal users.

Data were collected using an online structured questionnaire administered between December 2025 and February 2026. The questionnaire consisted of items measuring Information Quality, Trust and Credibility Signals, Platform Quality and Usability, Security and Privacy Protection, Risk Perception Reduction, Communication and Responsiveness, Governance and Ethical Transparency, and Consumer Confidence. All items were measured using a five-point Likert scale ranging from strongly disagree (1) to strongly agree (5). Participation was voluntary, anonymous, and based on informed consent.

A total of 200 survey invitations were distributed, of which 163 valid individual responses were received, yielding a response rate of 81.5%. For a study examining seven causal conditions, this sample size is methodologically robust for fsQCA, as it enables sufficient configurational diversity, supports appropriate frequency thresholds in truth-table construction, and enhances analytical stability. The fuzzy-set qualitative comparative analysis was conducted using fsQCA 4.1 (Windows version), developed by Charles C. Ragin. The software implements the Quine–McCluskey algorithm to generate complex, intermediate, and parsimonious solutions (43).

## 4 Results

This study uses fsQCA, a set-theoretic, configurational, and asymmetric modelling technique used to analyse complex causality with outcome CONF (Consumer Confidence) as the dependent variable, while seven conditions, INFQ, TRUS, USAB, SECP, RISK, COMM, and GOVE, are conditions or independent variables. The algorithm used for the modelling is Quine–McCluskey, with consistency and frequency cutoffs of 0.85 and 3, respectively.

### 4.1 Necessary condition analysis

Table 3 presents the results of the necessary condition analysis. None of the examined conditions reaches the conventional fsQCA consistency threshold of 0.90, indicating that no single factor can be considered necessary for achieving high consumer confidence in medical tourism portals. Although the absence of trust signals (∼TRUS; consistency = 0.851) and the absence of risk reduction (∼RISK; consistency = 0.821) exhibit relatively higher consistency values, they remain below the recommended threshold and therefore do not constitute evidence of necessity. Overall, the findings support the configurational premise of the study that consumer confidence emerges from multiple combinations of informational, technological, relational, and institutional factors rather than from any single dominant antecedent. This result is consistent with the principles of equifinality and conjunctural causation and justifies the subsequent sufficiency analysis.

**Table 3:** Summary of the necessary condition analysis.

Condition	Consistency	Coverage	Condition	Consistency	Coverage
INFQ	0.699364	0.641597	**∼INFQ**	0.740919	0.610386
TRUS	0.558869	0.654203	**∼TRUS**	0.851166	0.587169
USAB	0.682827	0.644735	**∼USAB**	0.787421	0.632565
SECP	0.713639	0.665218	**∼SECP**	0.742474	0.603100
RISK	0.661767	0.695690	**∼RISK**	0.820919	0.606897
COMM	0.674064	0.656164	**∼COMM**	0.772015	0.604739
GOVE	0.721131	0.635605	**∼GOVE**	0.743039	0.635440

### 4.2 Truth table analysis

Next, the fuzzy truth table was constructed to represent all logically possible configurations of the seven causal conditions. Given seven antecedent conditions, the total number of logically possible configurations equals 128. A frequency cutoff of 3 cases per configuration and a consistency threshold of 0.85 were applied to ensure that only empirically relevant and highly consistent configurations are retained for Boolean minimization. The truth table analysis revealed a limited number of empirically meaningful configurations meeting the consistency criterion, indicating that consumer confidence is associated with relatively specific and stringent combinations of conditions.

### 4.3 Sufficiency analysis

Boolean minimization using the Quine–McCluskey algorithm produced complex, intermediate, and parsimonious solutions. Out of these solutions, the complex solution and intermediate solution give the same solution, indicating the robustness of the solution. As suggested in the literature, we have discussed the intermediate solution in this study next. The sufficiency analysis identifies the distinct configurational pathways (S1 to S4) leading to high consumer confidence (CONF) in medical tourism portals, Table 4. The overall solution exhibits a coverage of 0.60 and a consistency of 0.77, indicating that approximately 60% of high-confidence cases are explained by the identified configurations. Although the overall consistency indicates moderate explanatory strength, it remains acceptable for exploratory fsQCA research involving complex socio-technical phenomena characterized by multiple interacting antecedents. The result suggests that consumer confidence is shaped by diverse configurational mechanisms rather than a single dominant causal structure.

**Table 4:** Summary of the intermediate solutions.

Conditions	S1	S2	S3	S4
INFQ	1	1	1	−1
TRUS	0	1	−1	−1
USAB	−1	−1	−1	1
SECP	−1	−1	1	1
RISK	1	1	−1	1
COMM	−1	−1	−1	−1
GOVE	−1	0	−1	1
Raw Coverage	0.38	0.32	0.38	0.34
Unique Coverage	0.02	0.01	0.02	0.01
Consistency	0.86	0.86	0.85	0.88

Next, we will explain the four pathways identified in this study:

**Pathway 1:** INFQ * ∼USAB * ∼SECP * RISK * ∼COMM * ∼GOVE

This configuration indicates that high consumer confidence emerges when strong information quality (INFQ) and effective risk mitigation (RISK) are present, even in the relative absence of usability, security mechanisms, communication support, and governance transparency. Substantively, this pathway reflects a cognitive assurance mechanism. In high-risk cross-border healthcare contexts, consumers appear to prioritize clear, reliable, and comprehensive information, as well as reduced uncertainty about treatment outcomes, pricing, and procedural risks. When these cognitive assurances are sufficiently strong, experiential and institutional platform features become less critical. This finding underscores the central role of informational clarity and uncertainty reduction in shaping confidence in medical tourism decisions.

**Pathway 2:** INFQ * TRUS * ∼USAB * ∼SECP * RISK * ∼COMM

This configuration shows that high consumer confidence emerges when strong information quality (INFQ), formal trust signals (TRUS), and effective risk mitigation (RISK) coexist, even when usability, security features, and communication responsiveness are relatively weak. This pathway represents a substantive trust-based assurance logic. Unlike the earlier interpretation, trust signals are present here and work synergistically with high-quality information and reduced uncertainty. Together, these elements provide cognitive and reputational reassurance sufficient to sustain consumer confidence, even if the platform experience (usability) and institutional infrastructure (security mechanisms) are less developed. This suggests that confidence can be built through a combination of informational credibility and formal legitimacy cues, without requiring strong interactive or technological sophistication.

**Pathway 3:** INFQ * ∼TRUS * ∼USAB * SECP * ∼RISK * ∼COMM * ∼GOVE

This configuration indicates that strong information quality (INFQ) combined with robust security and privacy protection (SECP) can generate high consumer confidence, even when trust signals, usability, governance transparency, communication support, and explicit risk-reduction mechanisms are weaker. This pathway reflects a security-based institutional substitution mechanism in which visible data protection and privacy safeguards compensate for weaker reputational and relational cues. In digital healthcare environments, the protection of sensitive medical data can function as a primary institutional anchor of trust, independently sustaining confidence in the absence of broader governance or communication support.

**Pathway 4:** ∼INFQ*∼TRUS*USAB*SECP*RISK*∼COMM*GOVE

This configuration indicates that high consumer confidence can emerge even when information quality and formal trust cues are relatively weaker, provided that usability (USAB), security/privacy protection (SECP), risk mitigation (RISK), and governance transparency (GOVE) are strong, while communication responsiveness (∼COMM) is not prominent. Substantively, this pathway reflects an institutional-experiential assurance mechanism: confidence is constructed through a combination of functional platform performance (ease of navigation and comparison), strong safeguards for sensitive data, credible governance and accountability structures, and reduced perceived uncertainty, together compensating for weaker informational depth and weaker symbolic trust cues. The presence of ∼COMM indicates that, in this pathway, confidence does not rely heavily on interactive responsiveness; instead, structural assurances and smooth user experience appear sufficient.

#### 4.3.1 Analysis of coverage and consistency

Although all four retained configurations exhibit high consistency (≥0.85), they differ in their empirical relevance as indicated by raw and unique coverage. Raw coverage reflects the proportion of high consumer confidence cases explained by each pathway, whereas unique coverage indicates the proportion of cases exclusively explained by a specific configuration. Among the four pathways, Pathway 3 demonstrates the highest raw coverage (0.384) and the highest unique coverage (0.0220), indicating that it explains the largest share of high-confidence cases and contributes the greatest independent explanatory power. This suggests that the combination of strong information quality and security/privacy protection, despite weaker governance, communication, and formal trust signals, represents the most empirically prevalent mechanism of confidence formation in the sample. In contrast, Pathway 4 exhibits the highest consistency (0.8827), indicating the strongest subset relationship with the outcome. While it explains a slightly smaller proportion of cases (raw coverage = 0.341), it represents the most internally coherent and reliable configuration. Taken together, Pathway 3 can be considered the empirically dominant pathway in terms of explanatory breadth, whereas Pathway 4 is the most robust in terms of configurational strength.

**Figure 3:**
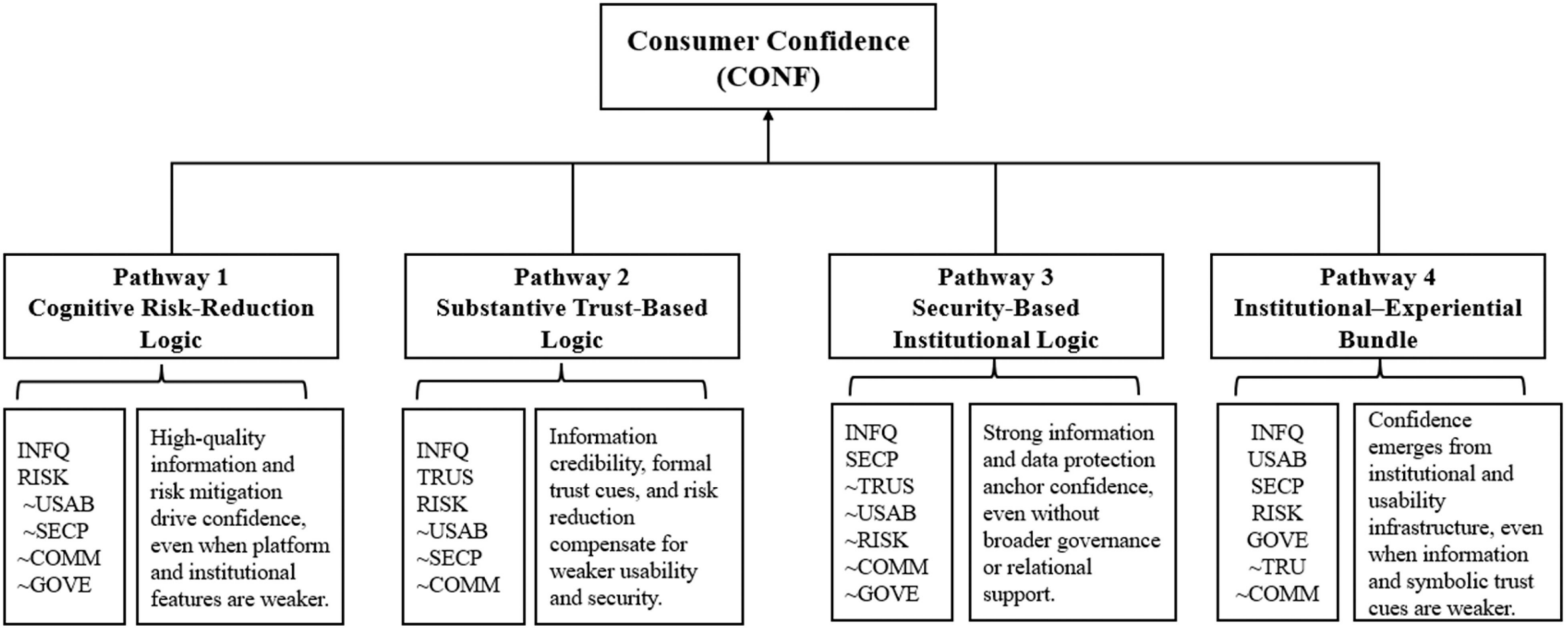
Configurational pathways leading to consumer confidence.

Figure 3 illustrates four configurational pathways leading to consumer confidence in medical tourism portals. Overall, the four pathways demonstrate equifinality: consumer confidence in medical tourism portals is achieved through multiple distinct combinations rather than a single dominant driver. Two pathways (1 and 2) emphasize cognitive assurance via information quality and risk mitigation, with Pathway 2 additionally highlighting the reinforcing role of trust/credibility cues. In contrast, Pathways 3 and 4 illustrate institutional assurance mechanisms, where security/privacy protection and governance transparency (and, in Pathway 4, usability) compensate for weaker information quality or trust cues. Collectively, the findings underscore the configurational and compensatory nature of digital trust formation in high-risk, cross-border healthcare decision environments.

## 5 Discussions

This study set out to examine how different combinations of platform-level and institutional factors generate consumer confidence (CONF) in medical tourism portals. By adopting a configurational lens through fsQCA, the findings move beyond the dominant linear assumptions in prior research (19, 41) and demonstrate that digital trust in cross-border healthcare is equifinal, compensatory, and asymmetric. Overall, four distinct pathways (S1–S4) explain 60% of high-confidence cases, reinforcing the proposition that consumer confidence in medical tourism portals does not arise from a single dominant driver, but from multiple interacting conditions. This insight directly responds to the research question and advances the literature on digital trust in high-risk healthcare ecosystems.

### 5.1 Challenging linear trust assumptions

The necessary condition analysis revealed that no single factor reached the 0.90 consistency threshold for necessity. This contrasts with earlier tourism and healthcare trust studies that implicitly treat constructs such as trust signals or information quality as foundational or universally required (31, 34). Interestingly, the absence of formal trust signals (∼TRUS) and the absence of explicit risk mitigation (∼RISK) showed relatively high consistency values. This does not imply that lower trust enhances confidence, but rather that high-confidence cases frequently coexist with partial non-membership in these sets. In other words, consumer confidence can be achieved even when traditional trust badges or explicit risk-reduction features are not dominant, provided other institutional or experiential mechanisms compensate.

This finding aligns with extant literature, which argues that accreditation and regulatory signals reduce but do not eliminate uncertainty in cross-border healthcare (9, 10). The present study extends this perspective by empirically demonstrating that accreditation or trust signals are not structurally indispensable when alternative assurances are present.

### 5.2 Primacy of information and risk reduction

Two pathways (S1 and S2) highlight the central role of Information Quality (INFQ) and Risk Perception Reduction (RISK) in generating confidence. This supports prior literature emphasizing the importance of transparent, comprehensive, and up-to-date medical information in shaping trust (30, 33). Medical tourism inherently involves high uncertainty regarding clinical outcomes, costs, and follow-up care (11, 13). Therefore, high-quality information and explicit uncertainty reduction mechanisms function as cognitive anchors. Pathway 2 further demonstrates that when Trust & Credibility Signals (TRUS) are added to strong information and risk mitigation, confidence emerges even if usability and security features are weaker. This aligns with relationship marketing research (34), which suggests that reputational and symbolic trust cues reinforce behavioural intention. However, the configurational insight is crucial: trust signals alone are insufficient. They must co-occur with strong informational clarity and reduced perceived risk. This nuanced finding refines prior linear models that position trust signals as independent predictors of intention (41).

### 5.3 Security as a core trust anchor

Pathway 3 reveals that Information Quality (INFQ) combined with Security & Privacy Protection (SECP) can generate high confidence even in the absence of strong governance signals, communication responsiveness, and formal trust badges. This finding strongly resonates with Guha (33), who highlights privacy concerns as a key determinant of digital engagement in medical tourism contexts. Given the sensitive nature of medical data, visible data protection measures appear to serve as institutional trust anchors. Moreover, this pathway reflects the growing importance of digital infrastructure in shaping healthcare trust ecosystems (6, 7). In digitally mediated healthcare, patients may interpret robust privacy mechanisms as indicators of professionalism and reliability, thereby compensating for weaker symbolic or relational trust cues. Notably, Pathway 3 has the highest raw and unique coverage, suggesting that security-based confidence formation is empirically dominant in the sample. This is particularly relevant in regions where cross-border legal harmonization remains uneven (9).

### 5.4 Governance and usability as compensatory drivers

Pathway 4 demonstrates that strong Usability (USAB), Security (SECP), Risk Mitigation (RISK), and Governance Transparency (GOVE) can compensate for weaker information quality and formal trust signals. This pathway reflects an institutional-experiential trust mechanism. Janiak & Kozłowska-Adamczak and Yazdani et al. show that usability influences perceived credibility and satisfaction in tourism portals (31, 35). The present study extends this to medical tourism, showing that seamless navigation and comparison tools function as experiential signals of professionalism. Additionally, governance transparency, such as clear complaint policies, regulatory alignment, and visible ethical commitments, aligns with literature (32, 38), who emphasize regulatory and ethical structures in medical tourism ecosystems. Importantly, this pathway suggests that institutional legitimacy and functional efficiency together can offset weaker informational depth. This complements Turner's assertion that governance structures enhance confidence but adds configurational nuance: governance works synergistically with usability and security, not in isolation (10).

Beyond institutional and platform-based trust mechanisms, consumer confidence may also be influenced by social validation obtained through online patient communities and social media platforms. Digital patient communities such as PatientsLikeMe, online health forums, social media groups, and patient review platforms enable prospective medical tourists to access experiential knowledge shared by individuals who have undergone similar treatments. These communities reduce information asymmetry by providing first-hand accounts of treatment experiences, provider interactions, recovery outcomes, and destination-specific challenges. Such peer-generated content can complement formal trust signals such as accreditation and governance mechanisms by offering social proof and perceived authenticity. In high-risk healthcare decisions, consumers often rely on both institutional assurances and experiential evidence when evaluating providers and destinations. Consequently, digital patient communities may function as an additional trust-building mechanism within the broader medical tourism ecosystem and represent an important area for future configurational research.

Beyond platform-level characteristics, trust formation in medical tourism is also influenced by the broader healthcare system within which providers operate. Strong health systems contribute to consumer confidence through robust regulatory oversight, accreditation frameworks, patient safety mechanisms, continuity-of-care arrangements, and effective coordination among healthcare stakeholders. For international patients, confidence is often shaped not only by trust in an individual provider or portal but also by perceptions of the destination country's healthcare infrastructure, quality standards, emergency response capabilities, and post-treatment support systems. Consequently, digital trust in medical tourism portals should be viewed as embedded within larger health system structures that provide institutional legitimacy, accountability, and long-term assurance for cross-border healthcare decisions.

## 6 Conclusions

This study advances understanding of consumer confidence in medical tourism portals by adopting a configurational perspective using fuzzy-set Qualitative Comparative Analysis (fsQCA). The findings demonstrate that consumer confidence does not depend on any single dominant factor but emerges through multiple equifinal combinations of information quality, security and privacy protection, governance transparency, usability, trust signals, and risk mitigation.

The results reveal the presence of compensatory mechanisms, whereby strengths in certain informational, technological, or institutional conditions can offset weaknesses in others. Four distinct configurational pathways were identified, highlighting the complex and non-linear nature of digital trust formation in medical tourism environments. These findings support the principles of equifinality and conjunctural causation and demonstrate the value of configurational approaches for examining trust-related phenomena in healthcare.

From a practical perspective, the study suggests that medical tourism portals should adopt a holistic trust-building strategy that integrates high-quality information, robust security measures, effective risk communication, user-friendly design, and transparent governance mechanisms. By moving beyond traditional linear models, this research provides both theoretical insights and actionable guidance for strengthening consumer confidence in digital healthcare ecosystems.

### 6.1 Limitations and future research directions

This study has several limitations. First, the sample comprised healthcare portal users rather than confirmed medical tourists; therefore, perceptions of consumer confidence may differ from those of individuals with actual cross-border treatment experience. Future research should validate the findings using samples of confirmed medical travellers and compare pre-travel confidence with post-treatment outcomes. Second, the cross-sectional design and reliance on self-reported measures limit causal inference and may be subject to response bias. Longitudinal studies incorporating behavioural indicators, such as booking intentions, platform usage data, or actual travel decisions, could provide stronger validation. Third, cultural and regional differences were not explicitly examined and may influence trust formation in medical tourism contexts. Future studies could explore cross-country variations and incorporate cultural dimensions as configurational conditions.

Finally, the overall solution consistency and coverage indicate that additional factors may contribute to consumer confidence. Future research may extend the model by examining the influence of destination reputation, prior healthcare experience, brand image, AI-enabled personalization, social media engagement, and online patient communities. Further studies could also strengthen methodological transparency by reporting additional fsQCA diagnostics, exploring pathways leading to low consumer confidence (∼CONF), and comparing configurational findings with SEM-based approaches. Emerging technologies such as blockchain-based verification, AI-driven risk assessment, and digital health passports also represent promising directions for future research.

## Data Availability

The datasets presented in this study can be found in online repositories. The names of the repository/repositories and accession number(s) can be found below: https://doi: 10.6084/m9.figshare.32101600.
